# A system-level metastable model of cancer evolution: integrating replication stress, cell cycle deregulation and chromosomal instability

**DOI:** 10.1080/07853890.2026.2620184

**Published:** 2026-01-28

**Authors:** Dongjing Ma, Jinbin Wang, Yicong Chen, Ling Yao, Qianya Wei, Jianjun Wu

**Affiliations:** School of Public Health, Gansu University of Chinese Medicine, Lanzhou, China

**Keywords:** Metastable state, chromosomal instability (CIN), replication stress (RS), cell cycle deregulation (CCD), therapy resistance

## Abstract

**Introduction:**

Cancer cell proliferation occurs within the context of persistent genomic instability. In this review, we propose the RS–CCD–CIN axis as a systems-level framework in which replication stress (RS), cell cycle deregulation (CCD) and chromosomal instability (CIN) form an interdependent triad that shapes tumour evolution. This axis represents a constrained metastable state in which genomic instability is tolerated and buffered. The objective of this review is to synthesize the current understanding of how the RS–CCD–CIN axis contributes to tumour heterogeneity, adaptability and therapy response.

**Discussion:**

Evidence indicates that RS, CCD and CIN operate as a dynamic, interconnected network rather than as independent processes. Replication stress induces DNA damage and mutagenesis, while partial checkpoint disruption permits cells with unresolved lesions to proliferate. Chromosomal instability generates both structural and numerical alterations, contributing to intratumoural heterogeneity. Together, these processes facilitate adaptation to environmental and therapeutic pressures. Extrachromosomal DNA, micronuclei formation and cytosolic DNA signalling, including the cGAS–STING pathway, connect genomic instability to adaptive responses and immune modulation. Single-cell and spatial profiling reveal temporal and spatial variability in RS, CCD and CIN states, highlighting the limitations of static biomarkers. Therapeutically, targeting individual components often yields limited durability, whereas approaches that simultaneously perturb multiple aspects of the RS–CCD–CIN axis may improve clinical outcomes.

**Conclusions:**

This review highlights the RS–CCD–CIN axis as a fragile and metastable architecture that supports cancer evolution, while also being susceptible to collapse. A deeper understanding of this interconnected framework may inform the development of therapeutic strategies and enhance the management of resistance.

## Introduction

Cancer cells exhibit an extraordinary capacity to survive and evolve despite experiencing high levels of genomic instability [1,2]. Rather than succumbing to extensive DNA damage, cancer cells can exist in a metastable state, defined as a non-equilibrium yet viable condition characterized by persistent genomic stress that is actively mitigated under specific cellular contexts [3,4]. In this review, metastability is utilized not merely as a descriptive metaphor but as a concept grounded in systems-level dynamics. This framework illustrates how cancer cells maintain a delicate balance between adaptive evolution and catastrophic collapse.

Importantly, metastability does not indicate maximal stress tolerance or extreme genomic instability; instead, it describes a constrained state maintained within a narrow viability window [5]. Central to this state is a coordinated interplay among replication stress (RS), cell cycle deregulation (CCD) and chromosomal instability (CIN) [6,7]. This triad constitutes a dynamic network that constrains the accumulation and distribution of genomic variation, enabling cancer cells to tolerate ongoing instability while preserving proliferative capacity and evolutionary potential.

RS, CCD and CIN represent the three principal challenges that cancer cells must navigate during tumour progression. Replication stress arises from obstacles to DNA replication, including stalled forks, DNA lesions and topological constraints, which can lead to fork collapse and the accumulation of mutations [8,9]. Cell cycle deregulation – often driven by mutations in key regulators such as *TP53*, *RB1* and *CDKs* – disrupts checkpoint control, enabling cells with damaged or incompletely replicated DNA to bypass critical surveillance mechanisms [10,11]. Chromosomal instability, characterized by recurrent structural and numerical chromosomal aberrations, promotes dynamic genome remodelling and heterogeneity [12]. While each of these factors contributes independently to tumour evolution, their synergistic interactions underpin the metastable nature of cancer cells.

The current literature predominantly examines RS, CCD and CIN as isolated phenomena. However, this fragmented approach fails to capture the system-level dynamics through which cancer cells integrate these mechanisms to construct a stress-buffered adaptive architecture. A critical gap persists in our understanding of how cancer cells dynamically regulate the interplay between RS, cell-cycle deregulation and CIN. This regulation is essential for sustaining short-term viability without precipitating catastrophic genome failure [13]. Furthermore, it is crucial to identify the conditions under which this metastable regime contributes to therapeutic resistance and immune modulation.

In this context, we use the term ‘metastable’ to denote a specific dynamical regime rather than a descriptive metaphor or a static clinical phenotype. Metastability refers to a non-equilibrium yet viable state in which cancer cells experience persistently elevated replication-associated stress, partially compromised cell-cycle checkpoint control and ongoing CIN, while remaining capable of sustained proliferation. Crucially, this state is characterized not by the extreme activation of any single process, but by the dynamic interplay among RS, cell-cycle deregulation and CIN [14]. This interplay allows for the transient buffering of perturbations in one dimension by compensatory responses in the others. When this coupling is maintained within a narrow range, genomic instability is tolerated and repurposed as an evolutionary substrate. However, when this range is exceeded or the coupling is disrupted, the system transitions abruptly towards catastrophic collapse. This definition distinguishes metastability from generalized stress tolerance or static genomic instability by emphasizing constrained viability, reversibility and proximity to a failure threshold [15].

Given the integrative nature of this framework, it is essential to delineate the evidentiary scope and causal intent of the arguments presented in this review. Throughout the manuscript, interactions within the RS–CCD–CIN axis are examined at various levels of empirical support. In instances where causal directionality is well established, claims are substantiated through genetic perturbation, pharmacological intervention and *in vivo* validation. Conversely, in other cases, relationships are characterized as conditional or bidirectional couplings, which depend on tumour context, genetic background or temporal dynamics, rather than being represented as linear cause–effect chains. Finally, system-level concepts that synthesize multiple lines of evidence are explicitly framed as hypothesis-generating, aiming to organize existing observations and guide future experimental falsification, rather than asserting mechanistic completeness.

This review presents a systems biology model of metastability, which conceptualizes cancer cell fate as emerging from a dynamic RS–CCD–CIN axis. Rather than proposing a new tumour classification, this framework seeks to elucidate the dynamic processes by which cancer cells enter, sustain and exit metastable evolutionary states. We contend that cancer cells do not depend on a singular driver of adaptability; instead, they rely on a finely regulated network of interdependent processes that collectively maintain genomic instability within a tolerable threshold. This network is proposed to enhance cancer cell survival in the face of replicative, mitotic and environmental stresses. Additionally, under specific conditions, it may influence therapeutic adaptation and immune interactions.

By elucidating the tripartite feedback loops and compensatory mechanisms among RS, CCD and CIN, this model presents a cohesive conceptual framework for comprehending cancer evolution. Furthermore, it justifies the targeting of metastable collapse as a therapeutic strategy, shifting the emphasis from single-pathway inhibition to the systemic disruption of the fragile genomic equilibrium inherent in cancer.

## Non-linearity and threshold behaviour in the RS–CCD–CIN axis

Framing cancer evolution within a metastable regime generates a set of empirically testable predictions that differentiate the RS–CCD–CIN framework from linear models of genomic instability or generic stress tolerance. Specifically, the relationship among RS, CIN and cellular fitness is expected to be fundamentally non-linear; moderate, sustained perturbations are predicted to promote adaptability, while exceeding a critical threshold leads to an abrupt loss of viability rather than a gradual decline [16–18]. The model also predicts a strong temporal dependence, indicating that identical genetic or pharmacological perturbations may either stabilize the metastable regime or induce collapse, contingent upon the cell-cycle phase, stress history and buffering capacity at the time of intervention. Notably, partial attenuation of one axis – such as a transient reduction of RS – may paradoxically enhance long-term resistance by stabilizing the coupled system instead of sensitizing it [19,20]. Finally, metastable tumours are predicted to display dynamic, oscillatory patterns of RS, CCD and CIN at the single-cell level over time, rather than remaining in fixed high or low instability states, a characteristic not anticipated by static genomic instability paradigms [21,22].

## Replication stress: the paradoxical engine of genomic catastrophe and evolutionary adaptation

**Figure 1.**
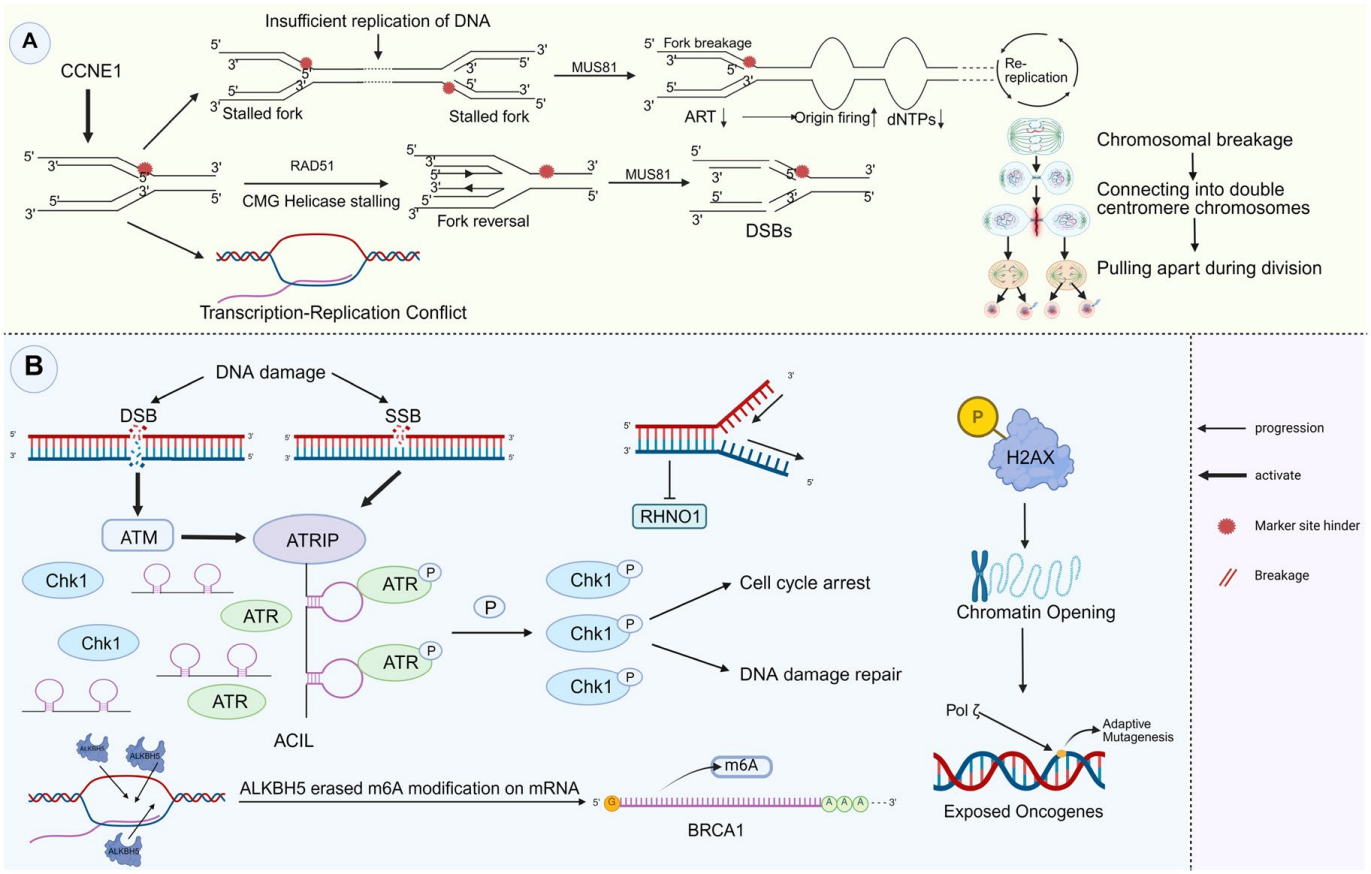
Mechanisms linking RS to genomic instability and adaptive survival. (A) *Structural instability*: High levels of replication stress, driven by CCNE1, overwhelm the mechanisms that protect replication forks. The processing of reversed replication forks by MUS81 leads to the formation of double-strand breaks (DSBs). In the absence of sufficient repair, these breaks contribute to breakage-fusion-bridge (BFB) cycles, resulting in complex karyotypic abnormalities, such as dicentric chromosomes. (B) *Molecular adaptation*: In addition to activating canonical checkpoints (ATM/ATR-Chk1), RS promotes evolutionary adaptation through two mechanisms: (1) epigenetic modulation, where R-loop formation recruits ALKBH5 to regulate BRCA1 via m6A demethylation; and (2) mutagenic tolerance, in which H2AX-mediated chromatin remodelling facilitates Pol ζ-dependent error-prone replication, thereby introducing driver mutations in response to stress.

RS in cancer cells is best conceptualized as a persistent, graded cellular state rather than a transient insult. In this context, intermediate and sustained levels of replication perturbation contribute to genetic diversification, while excessive or unbuffered stress leads to irreversible fork collapse and ultimately results in cell death [19]. Oncogene-induced hyperproliferation forces replication forks to traverse topological barriers such as heterochromatin and repetitive DNA. This mechanical conflict between the CMG helicase and nucleosome remodelling complexes leads to replication fork reversal and collapse [23]. Fork collapse generates structurally complex DNA intermediates that, depending on the competence of the checkpoint and the engagement of repair pathways, may lead to double-strand DNA breaks. These breaks can initiate breakage–fusion–bridge (BFB) cycles or alternative segregation outcomes, thereby contributing to heterogeneous chromosomal rearrangements in oncogene-rich regions in a context-dependent manner [24] (Figure 1(A)).

Within the evolutionary landscape of cancer, RS functions as a catalyst for adaptive mutagenesis. Sustained activation of the ATR–Chk1 pathway not only extends the cell cycle but also modifies chromatin accessibility through histone H2AX phosphorylation [25–27] (Figure 1(B)). This chromatin remodelling creates an open configuration around stalled replication forks, exposing nearby oncogenes such as *CCNE1*. When translesion synthesis polymerases such as POLζ resume DNA replication, their error-prone activity introduces driver mutations within temporally regulated replication domains [28]. This process may disrupt the boundaries of topologically associating domains (TADs), potentially exposing epigenetically silenced regions, such as tumour suppressor loci, to mutagenesis associated with RS under certain conditions. The resulting collapse of both genetic and epigenetic integrity enables cancer cells to acquire multiple classes of mutations within a single replication cycle. The complex interplay between RS and therapeutic resistance highlights the adaptive strategies that cancer cells use to manage genomic instability [29]. PARP inhibitors exacerbate replication fork stalling by inducing PARP trapping on DNA. However, prolonged treatment selects for subclones with impaired micronuclear envelope formation, allowing damaged DNA to escape encapsulation and persist in the cytoplasm [30]. These cells sequester damaged replication forks within micronuclei, where rupture of the nuclear envelope exposes DNA to the cytosol, thereby activating the cGAS–STING pathway [31]. This signalling can induce context-dependent outcomes: under acute activation, it promotes immunogenic type I interferon (IFN) responses, while sustained activation may drive prosurvival autophagy and engage NHEJ-mediated DNA repair [32], illustrating that cGAS–STING engagement can act both as a consequence and as a modulatory node influencing RS–CCD–CIN dynamics. Notably, RS induces transcription–replication conflicts that cause RNA polymerase II to stall ahead of the replication fork, resulting in the formation of stable RNA–DNA hybrids, known as R-loops [33]. These R-loops modulate the epigenetic flexibility of DNA repair pathways by recruiting demethylases such as ALKBH5, which removes m^6^A modifications from mRNAs encoding repair genes such as *BRCA1* [34,35]. This mechanism allows cancer cells to rapidly switch between DNA repair pathways in response to therapeutic pressure.

## Cell cycle deregulation (CCD): the subversion of temporal order in the evolutionary crucible of cancer

**Figure 2.**
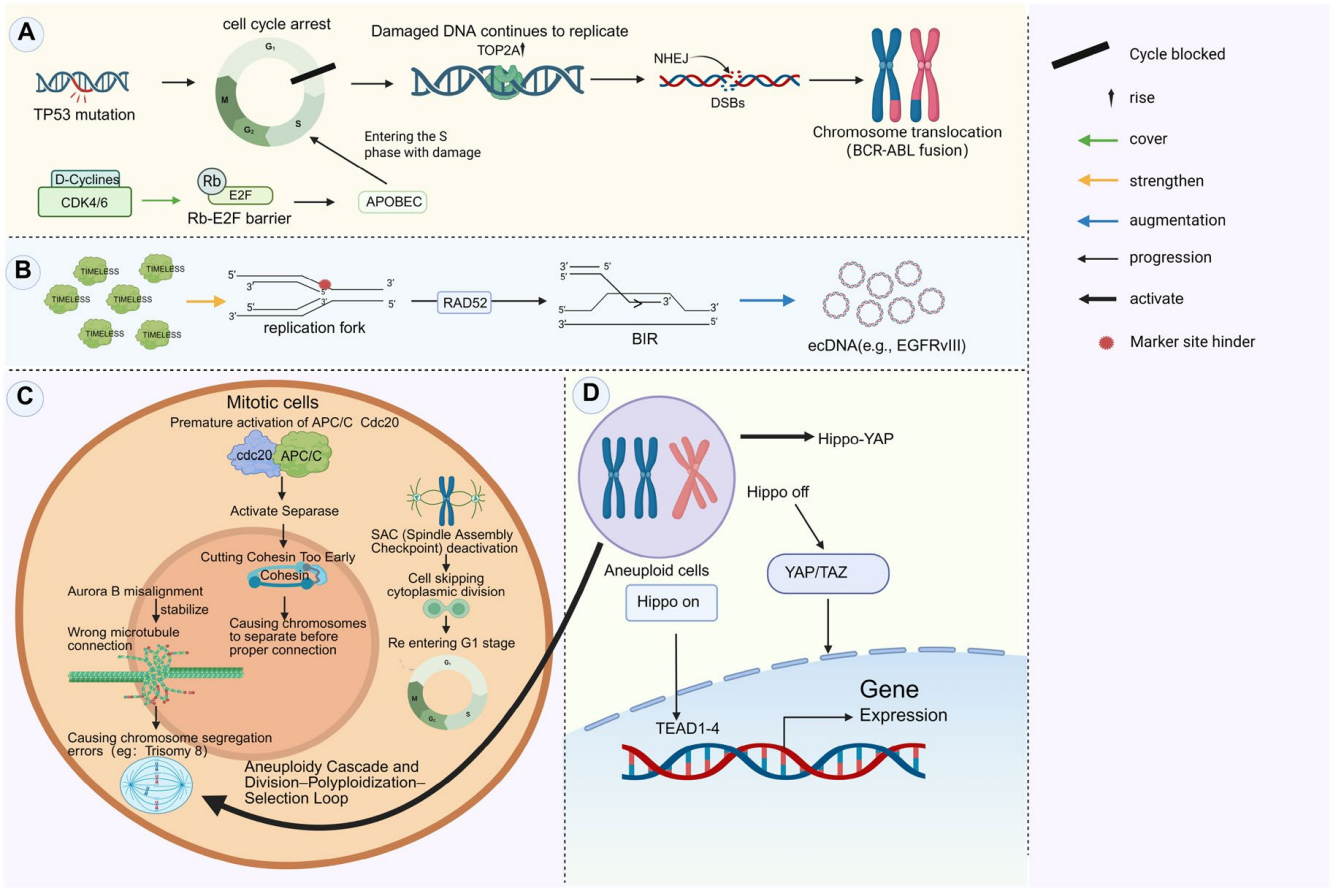
Mechanisms of CCD and therapeutic adaptation. (A) Erosion of the G1/S checkpoint. Mutations in TP53 and the activation of CDK4/6 allow cells to bypass the Rb–E2F barrier, promoting replication even in the presence of DNA damage. This results in TOP2A-mediated DSBs, which are improperly repaired by non-homologous end joining (NHEJ), leading to translocations such as BCR–ABL. (B) Replication stress and ecDNA. Stalled replication forks are stabilized by TIMELESS and are subsequently restarted through RAD52-dependent BIR. This mechanism produces circular ecDNA, such as EGFRvIII. (C) Dysregulation of mitosis. Premature activation of the APC/C^Cdc20^ complex leads to early cleavage of cohesin, a phenomenon referred to as ‘temporal hijacking.’ Coupled with misalignment of Aurora B and failure of the SAC, this results in mitotic slippage, which can cause polyploidy and aneuploidy, exemplified by Trisomy 8. (D) The polyploidy–resistance loop. In polyploid cells, inactivation of the Hippo pathway activates nuclear YAP/TAZ–TEAD signalling. Together with WGD and resistance mutations, such as deletion of ERCC1, this facilitates evasion of contact inhibition and contributes to therapy resistance.

In the context of cancer metastability, CCD signifies a partial erosion of temporal checkpoint control rather than a complete abrogation of these checkpoints. This allows for the continued progression of the cell cycle despite the presence of genomic lesions, without immediately precipitating mitotic catastrophe [36]. At its core, CCD arises from the breakdown of key cell cycle checkpoints: *TP53* mutations impair the G1/S transition, whereas aberrant CDK4/6–cyclin D activation bypasses the Rb–E2F regulatory barrier [37,38]. This allows replication to proceed over unrepaired DNA lesions, such as those induced by APOBEC enzymes. Aberrant activity of TOP2A at replication forks generates double-strand breaks (DSBs), which are misrepaired by non-homologous end joining (NHEJ), leading to chromosomal translocations and oncogenic fusions such as *BCR–ABL* [39] (Figure 2(A)).

Cell cycle deregulation not only drives genomic instability but also contributes to therapy resistance. In CCD-positive cells, taxane-induced mitotic arrest can promote genome remodelling. Meanwhile, stabilized replication forks, which are maintained by TIMELESS, may occasionally be restarted through RAD52-dependent break-induced replication (BIR) [15,40]. This process creates conditions that are conducive to the formation of ecDNA, including EGFRvIII [41,42] (Figure 2(B)).

Mitotic dysregulation represents the apex of genomic instability. Premature activation of APC/C^Cdc20^ triggers early cohesin cleavage by separase, resulting in chromosome missegregation before proper kinetochore attachment – a process referred to as ‘temporal hijacking’ [43]. Mislocalization of Aurora B kinase stabilizes incorrect kinetochore–microtubule attachments, promoting the persistence of selectively advantageous aneuploidies, such as trisomy 8. In parallel, partial inactivation of the spindle assembly checkpoint (SAC) permits mitotic slippage, allowing cells to bypass cytokinesis and re-enter G1 as polyploid cells. These tetraploid cells initiate an ‘aneuploidy cascade,’ generating subclonal diversity that surpasses the evolutionary potential of point mutations alone (Figure 2(C)).

Polyploid cells can circumvent contact inhibition by activating the Hippo–YAP pathway and may exhibit resistance to genotoxic agents, such as cisplatin, through whole-genome duplication (WGD) under specific conditions [44]. Critically, they retain key resistance mutations, such as *ERCC1* deletion (Figure 2(D)). This division–polyploidization–selection loop serves as a central driver of therapeutic adaptation [45].

Treatment strategies are shifting from inducing cell cycle arrest to targeting CCD-specific vulnerabilities. Although CDK4/6 inhibitors effectively induce G1 arrest, their efficacy is often bypassed through cyclin E–CDK2 activation or loss of *FBXW7*. New approaches focus on exploiting synthetic lethality at mitotic exit. For example, PLK1 inhibition disrupts checkpoint reset, resulting in chromosome missegregation and the loss of essential loci, such as the miR-15a/16-1 cluster, ultimately triggering apoptotic collapse [46]. In cells lacking G1 checkpoint control, ATR inhibitors exacerbate RS, leading to lethal transcription–replication collisions and R-loop accumulation during mitosis [47].

## Chromosomal instability: an important driving mechanism of tumour evolution, adaptation and resistance

**Figure 3.**
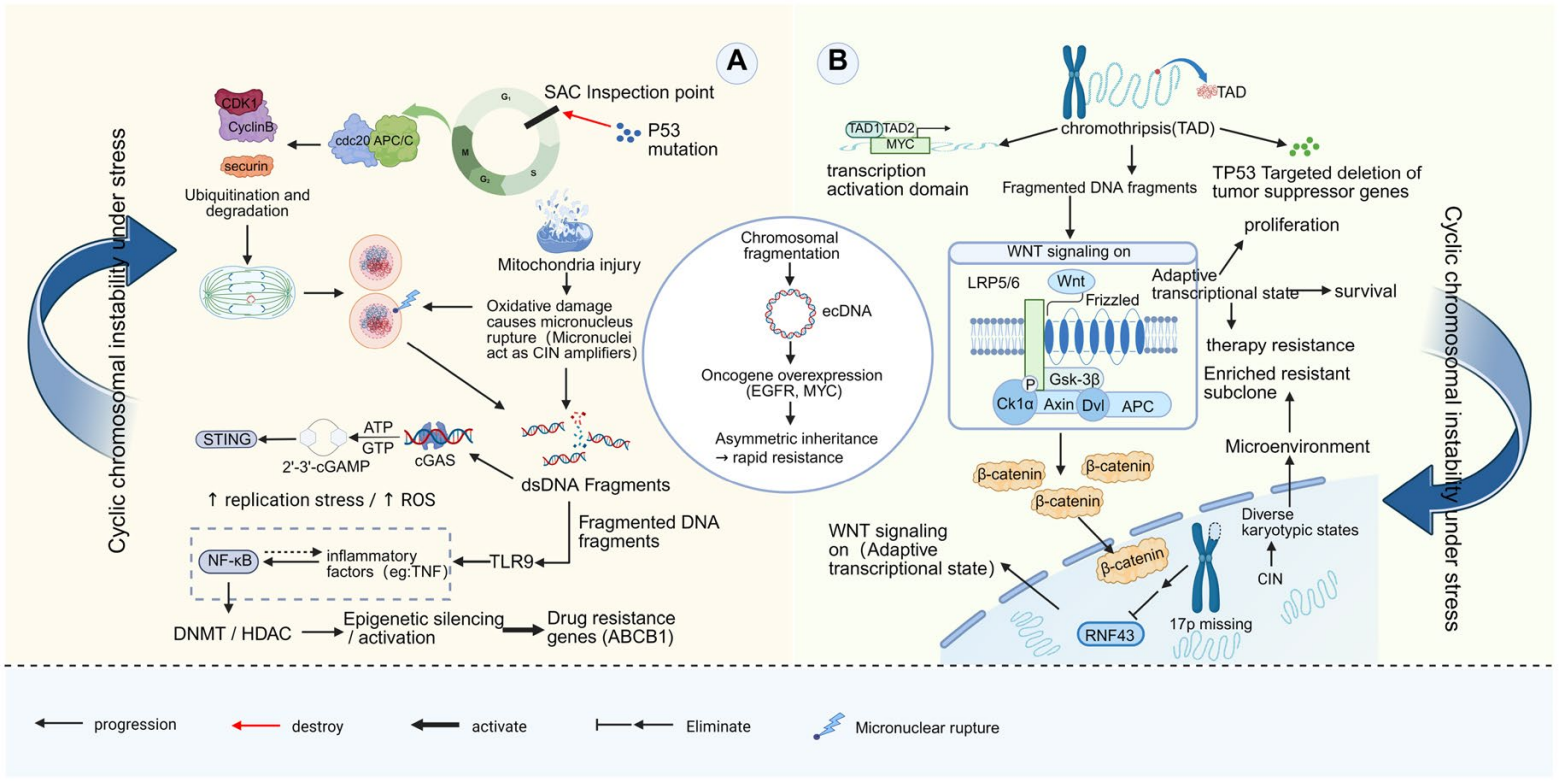
Cyclic CIN drives co-evolution of epigenetic reprogramming and adaptive resistance. (A) *The inflammatory–epigenetic axis*: Mitotic checkpoint failure leads to micronuclear rupture and cytosolic DNA exposure. This activates DNA sensing pathways (cGAS–STING and TLR9), triggering NF-κB-mediated inflammation. This signalling upregulates epigenetic modifiers (DNMTs/HDACs), leading to the silencing of drug metabolism genes and activation of efflux pumps like ABCB1. (B) *Karyotypic restructuring and signalling adaptation*: Chromosomal fragmentation generates ecDNA for rapid oncogene amplification (e.g. EGFR) and asymmetric inheritance. Concurrently, specific chromosomal losses, such as the 17p deletion, remove the WNT suppressor RNF43. This structurally activates WNT/β-catenin signalling, promoting cell survival and the expansion of resistant subclones.

Within a metastable evolutionary framework, CIN is characterized not solely by static aneuploidy, but by a continuous temporal variability in both chromosome number and structure. This variability generates fluctuating karyotypic states that can be selectively amplified or purged in response to stress [48,49]. This instability is initiated by a breakdown of mitotic checkpoint control [50–54]. Mutations in *TP53* compromise the G1/S checkpoint, narrowing the corrective window provided by the SAC and allowing cells to prematurely exit mitosis despite unresolved chromosomal segregation errors [55,56]. Micronuclei, formed as a consequence of chromosome missegregation, not only reflect segregation failure but also act as sites of extensive genomic damage [53,57,58]. The rupture of the micronuclear envelope exposes DNA to the cytosol, potentially activating the cGAS–STING pathway. This activation can lead to downstream inflammatory signalling and chromosomal fragmentation, which may vary depending on the cellular context, stress history and checkpoint integrity [59–61]. This fragmentation is not random; instead, it follows breakpoint patterns aligned with TAD boundaries. These patterns promote localized amplification of oncogenes such as MYC and selective loss of tumour suppressors such as TP53, reinforcing a self-perpetuating cycle of genomic disintegration [62–64] (Figure 3(A)).

From an evolutionary perspective, tumour adaptability mediated by CIN aligns with the ‘chromosomal ecological niche’ theory, which posits that each aneuploid karyotype gives rise to a distinct transcriptomic profile. For example, trisomy 21 induces IFN pathway activation through gene dosage effects, expanding the phenotypic repertoire available to cancer cells under microenvironmental stress [65]. Selection pressures from chemotherapy or targeted therapies can rapidly enrich subclones with specific patterns of chromosomal gains and losses. In ovarian cancer, for example, the polyploidy of chromosome 8 enhances resistance to hypoxic stress by increasing the dosage of glycolytic genes, whereas the deletion of 17p promotes WNT signalling by removing the E3 ubiquitin ligase RNF43 [66,67] (Figure 3(B)). Unlike point mutations, this chromosomal adaptation enables tumours to rapidly revise their karyotype architecture in response to therapy.

The mechanisms of drug resistance are closely tied to the coevolution of CIN and epigenetic reprogramming [68–71]. Nuclear DNA fragments generated by chromosomal breaks are detected by cytoplasmic DNA sensors such as TLR9, leading to activation of the NF-κB signalling pathway. This activation upregulates DNA methyltransferases (DNMTs) and histone deacetylases (HDACs), leading to widespread epigenetic disruption [72]. As a result, drug resistance-related genes such as ABCB1 become aberrantly activated, while genes involved in drug metabolism are silenced. CIN-induced ecDNA forms dynamic circular structures within the nucleus, often containing superenhancers that promote high-level expression of oncogenes, such as EGFR, and enable the rapid transmission of drug resistance through asymmetric segregation [73]. However, it is essential to acknowledge that similar effects of oncogene amplification and expression can occur through alternative genomic mechanisms. These mechanisms include BFB cycles, segmental amplifications and WGD, which may mimic the transcriptional outputs mediated by ecDNA and contribute similarly to cellular adaptation and therapeutic resistance [14,74].

The major challenge of tumour heterogeneity arises from a ‘quantized genomic landscape’ shaped by CIN. Within a single tumour, multiple karyotypic subclones can coexist, each occupying a distinct ecological niche enabled by aneuploidy-driven metabolic reprogramming, for example, chromosome 7 polysomy enhances glutamine metabolism to support clonal fitness [75]. These subclones maintain a dynamic equilibrium through cyclic chromosomal instability (cyclic CIN). Under therapeutic pressure, dominant clones may decline, allowing previously dormant subclones to regain evolutionary advantage through episodic bursts of CIN [76]. This chromosomal oscillation enables tumours to build a multilayered buffering system that operates across both spatial and temporal scales.

## Metastable collapse: critical disintegration of the cancer evolution engine

**Figure 4.**
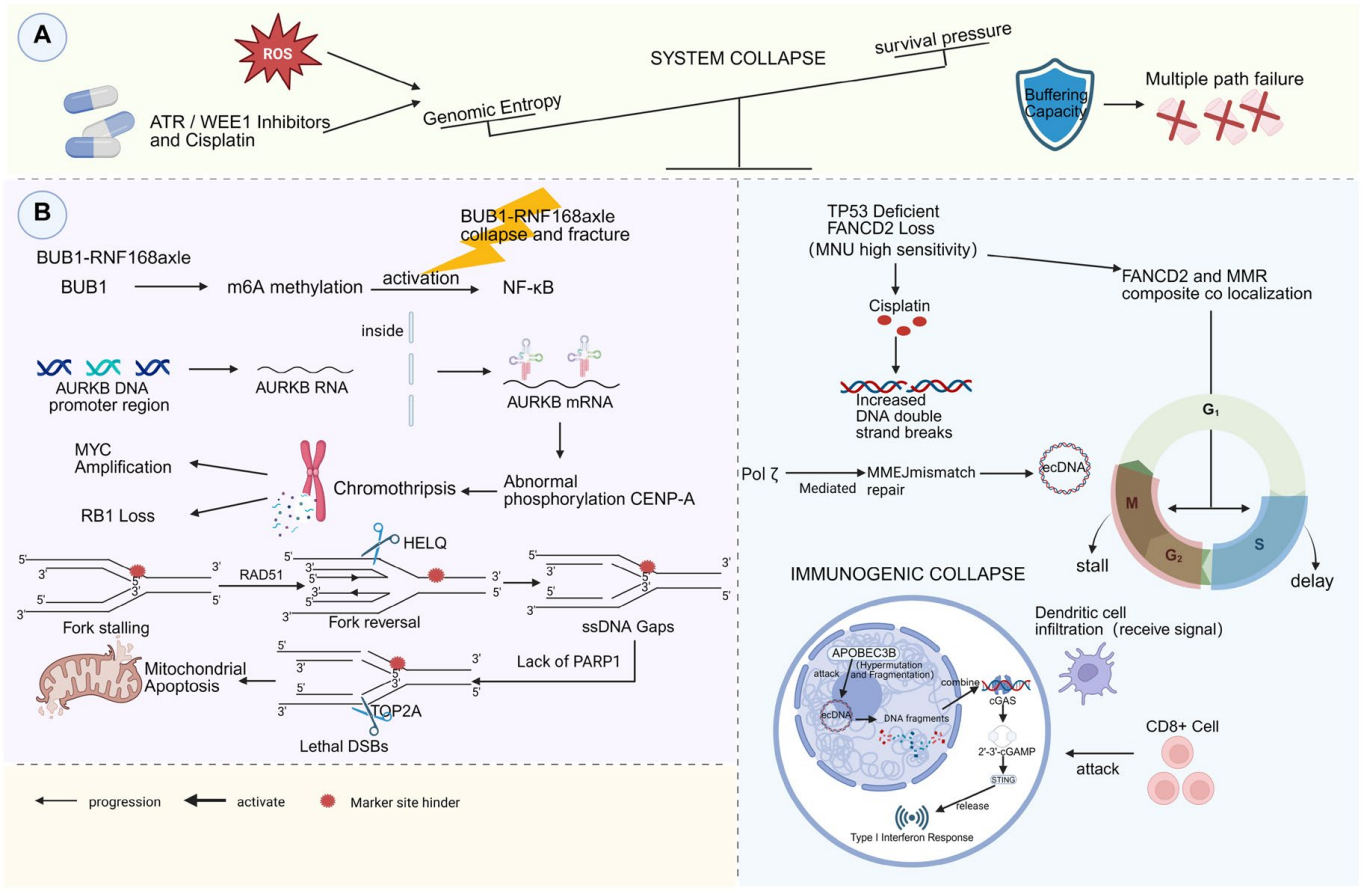
*Metastable collapse*: The critical disintegration of cancer evolution machinery. (A) *The tipping point of genomic entropy*: The schematic illustrates the metastable equilibrium as a balance between genomic entropy (driven by ROS and therapeutic agents like ATR/WEE1 inhibitors) and the system’s buffering capacity. System collapse occurs when entropy overwhelms the buffering mechanisms. (B) Multi-pathway mechanisms of system failure are characterized by distinct molecular catastrophes. On the left (replication and mitotic collapse), the breakdown of the BUB1–RNF168 axis and aberrant phosphorylation of AURKB drive chromothripsis. Concurrently, replication stress leads to the HELQ-mediated dismantling of reversed forks; in the absence of PARP1, TOP2A converts ssDNA gaps into lethal DSBs, triggering mitochondrial apoptosis. On the right (immunogenic collapse), in contexts deficient in TP53 and FANCD2, cisplatin-induced stress promotes POLθ-mediated MMEJ and the formation of ecDNA. These ecDNAs undergo hypermutation and fragmentation induced by APOBEC3B, effectively activating the cGAS–STING pathway and inducing a type I interferon response that recruits CD8+.

A critical implication of the metastable framework is that elevated RS or CIN alone is insufficient to define metastability. Specifically, when the buffering capacity is exhausted or the coupling between RS, CCD and CIN is disrupted, genomic instability transitions from being adaptive to acutely deleterious. The metastable state in cancer represents a delicate equilibrium between genomic entropy and survival constraints. Its collapse is triggered when this balance is disrupted by internal pressures or therapeutic intervention [77] (Figure 4(A)). Excess DNA generated by CIN can overwhelm the cGAS–STING pathway, shifting NF-κB-mediated survival signalling towards a type I IFN response. This transition is mediated through the cGAMP–STING–TBK1 signalling axis [78,79] (Figure 4(B)). Exposed chromatin fragments are taken up by dendritic cells, leading to antigen presentation and the activation of cytotoxic T-cell responses [80]. This cascade is particularly lethal in TP53-deficient lung squamous cell carcinoma. In these tumours, cisplatin-induced RS – intensified by loss of *FANCD2* – misdirects DNA repair towards POLθ-mediated microhomology-mediated end joining (MMEJ), leading to the formation of circular ecDNA. During mitosis, these ecDNAs are fragmented and hypermutated by APOBEC3B, generating immunogenic neoantigens that disrupt immune tolerance [81].

Collapse arises from the breakdown of entropy-buffering networks. When CIN exceeds the repair capacity of the BUB1–RNF168 axis, aberrant AURKB-mediated phosphorylation of CENP-A leads to chromosomal bridges that rupture during cytokinesis. This rupture triggers chromothripsis, resulting in oncogene amplification (e.g. MYC) and the loss of tumour suppressors (e.g. RB1) [82]. Moreover, deregulated RS induces RAD51-independent fork reversal. These reversed forks are dismantled by HELQ, resulting in single-stranded DNA (ssDNA) gaps [83]. PARP1 depletion depletes cellular NAD^+^ pools, leading to metabolic collapse. This process is accelerated by CCD, where sustained CDK2 activity drives mitotic entry despite unresolved damage. In the absence of PARP1, ssDNA gaps are converted into lethal DSBs through TOP2A-mediated cleavage, resulting in catastrophic replication–mitosis collisions [84,85].

Therapies designed to target metastable collapse aim to induce an ‘entropy overload,’ which represents a critical threshold where the rate of structural variation surpasses the proteostatic buffering capacity. Clinically, this state can be quantified by measuring elevated weighted genome integrity indices (WGII) and an increase in cytosolic dsDNA species. In ovarian cancer, the combination of ATR inhibitors, such as berzosertib, with WEE1 inhibitors, like adavosertib, may hinder the recovery of replication forks and reduce the duration of the G2/M phase. This creates conditions that may lead to premature entry into mitosis under conditions of high RS. The outcome of this combination treatment is highly dependent on factors such as dosing, timing and the specific cellular context [86,87]. During mitosis, fragments of unrepaired DNA can form ecDNA, which may activate the cGAS–STING pathway. This activation leads to the induction of type I IFN responses and promotes the priming of cytotoxic T-cells. In scenarios of acute or transient cGAS–STING activation, this mechanism transforms genomic instability into immunogenicity, thereby enhancing therapeutic vulnerability. Importantly, the magnitude and timing of cytosolic DNA accumulation, along with the burden of CIN, are critical determinants of this effect. Tumours characterized by high levels of CIN and rapid ecDNA turnover are more likely to engage in pro-immunogenic signalling, particularly when interventions are temporally coordinated [88,89]. This underscores that ecDNA-induced cGAS–STING activation can serve as a mechanistic readout of metastable collapse, provided that the intervention occurs within a temporal window dominated by acute signalling [90]. In EGFR-mutant lung cancer, combining osimertinib with MCL1 inhibitors impairs homologous recombination by promoting ALKBH5-mediated demethylation of BRCA1. Simultaneously, this combination lowers the threshold for mitochondrial apoptosis, resulting in enhanced tumour regression [91,92].

Ongoing clinical trials (e.g. NCT04267939) have demonstrated that combined *ATR* and *MCL1* inhibition in *TP53*-mutant tumours induces a coordinated collapse involving apoptosis, pyroptosis and immune-mediated clearance, as revealed by single-cell sequencing. These findings highlight a central paradox: the same genomic instability that fuels cancer evolution becomes a liability under targeted therapeutic pressure. By tipping this metastable balance, therapy can trigger catastrophic system failure – converting adaptability into vulnerability and driving tumours towards irreversible collapse.

## Clinical breakthrough: metastability collapse-driven precision treatment paradigm

The metastable model of cancer refers to a therapeutic strategy – shifting from single-pathway inhibition to coordinated disruption of a dynamic stress network. Traditional monotherapies, such as Aurora kinase or ATR inhibitors, often fail because of the entropy-driven adaptability of cancer cells. Suppressing CIN can paradoxically increase replication fork stability through *RAD51*, while blocking RS may inadvertently promote CCD-driven metastatic escape via CDK1–Rac1 signalling [93]. These responses reflect a dynamic redistribution of cellular stress mediated by epigenetic reprogramming. For example, KDM5A-driven histone demethylation facilitates rapid adaptation and contributes to therapeutic resistance [94].

To address this challenge, a strategy of systemic entropy intervention has emerged – targeting the interdependent axes of CIN, RS and CCD. For example, combining the Aurora A inhibitor alisertib with the ATR inhibitor berzosertib induces synthetic lethality in ovarian cancer. Alisertib disrupts kinetochore–microtubule attachments, inducing chromosomal misalignment and micronuclei formation – amplifying CIN. Moreover, berzosertib compromises replication fork protection, exacerbating RS and forcing unrepaired DNA into mitosis [95]. The synergy between ATR and Aurora A inhibition arises from temporal mismatch: ATR inhibitors stall replication forks, whereas Aurora A inhibitors accelerate mitotic entry, causing fatal replication–mitosis conflict. The clinical translation of these synergistic combinations is limited by a narrow therapeutic index, as ATR and CHK1 are vital for the function of normally proliferating tissues, including hematopoietic stem cells [96]. This necessitates the development of careful dosing strategies to balance efficacy and toxicity. Effective patient stratification requires biomarkers beyond TP53, such as CCNE1 amplification, baseline CIN metrics, ecDNA burden, RS markers (e.g. phosphorylated RPA32, γH2AX) and SLFN11 expression [97,98]. Tumours exhibiting high CCNE1 and elevated RS markers may preferentially respond to ATR plus CDK2 inhibition, while those with moderate CIN and significant ecDNA burden may show sensitivity to ATR plus Aurora A combinations [99]. Emerging protocols are investigating pulsed or sequential dosing schedules to leverage the differential recovery kinetics between tumour cells – operating near the threshold of replicative collapse – and normal tissues. The feasibility and efficacy of these dosing schedules remain context-dependent and are currently under evaluation in early-phase clinical trials. Clinical trials support this strategy [100]. In NCT04267939, TP53-mutant tumours treated with alisertib and berzosertib demonstrated a preliminary objective response rate (HR = 0.52). Single-cell analyses indicated features consistent with metastable collapse, including CIN, RS and CCD, which require validation in larger cohorts. Similarly, in NCT04586335, the combination of adavosertib (WEE1 inhibitor) and olaparib (PARP inhibitor) in BRCA-wild-type breast cancer resulted in increased chromothripsis and tumour mutational burden. While these findings provide mechanistic insights, they remain context-dependent and serve primarily as hypothesis-generating observations.

This paradigm marks a transition from targeting isolated vulnerabilities to exploiting systemic fragility. By collapsing the metastable architecture that sustains cancer evolution, precision therapy becomes a tool not only for control but also for conversion – transforming adaptive potential into therapeutic liability.

## The metastable frontier: decoding the tripartite nexus of replication stress, cell cycle deregulation and chromosomal instability in cancer evolution

**Figure 5.**
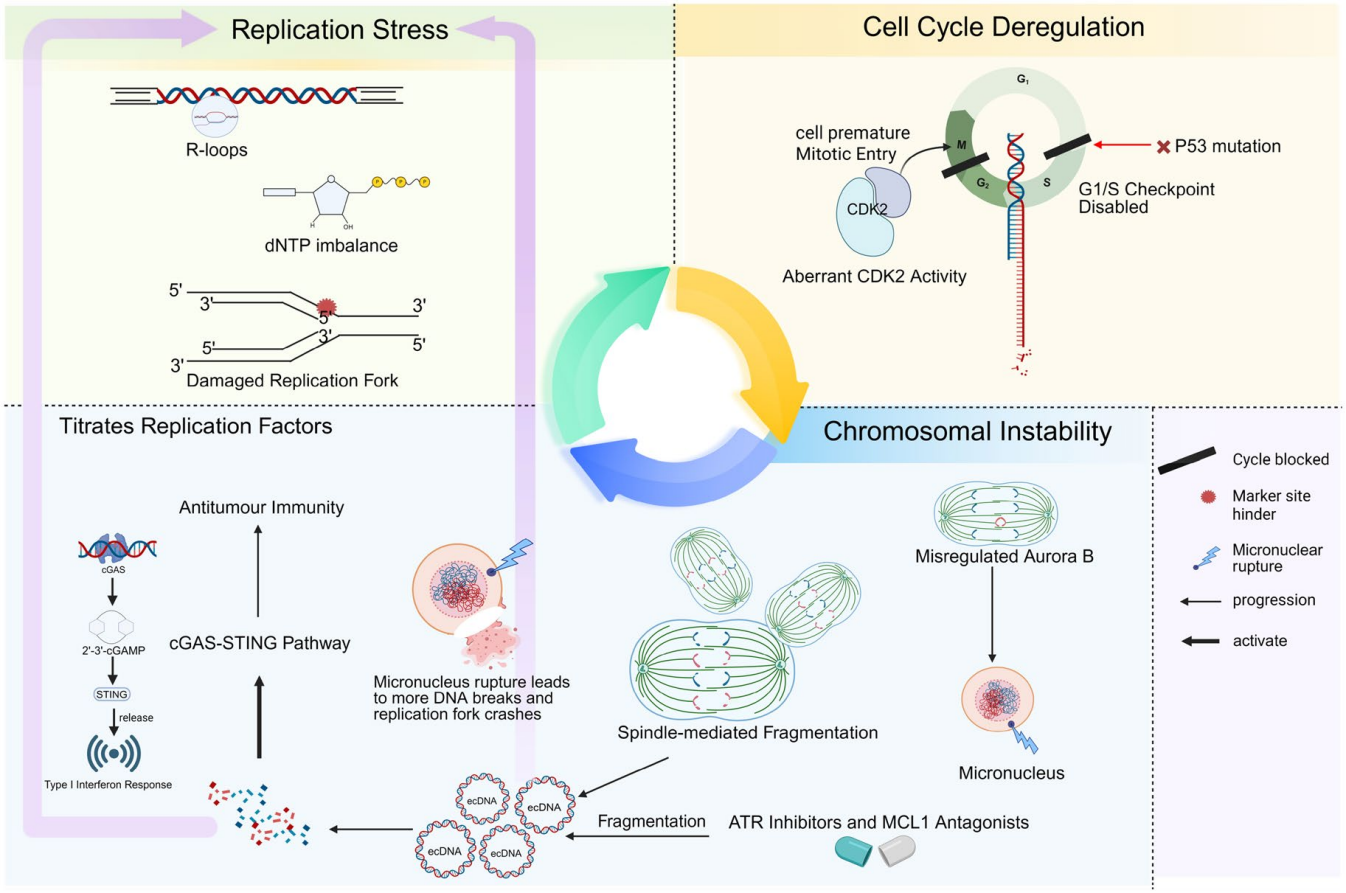
The metastable frontier: The tripartite nexus of RS, CCD and CIN. *Top left* (RS): Persistent replication stress disrupts genomic homeostasis through dNTP imbalance and R-loop accumulation, damaging replication forks. *Top right* (CCD): TP53 mutation disables the G1/S checkpoint. Coupled with aberrant CDK2 activity, this forces cells to enter mitosis prematurely with under-replicated DNA. *Bottom right* (CIN): Mitotic errors are propagated by misregulated Aurora B and spindle-mediated fragmentation, leading to micronucleus formation. *Bottom left* (feedback and therapy): The rupture of micronuclei and the accumulation of ecDNA create a feedback loop that titrates replication factors, further amplifying stress. Therapeutically, the combination of ATR inhibitors and MCL1 antagonists forces ecDNA fragmentation, activating the cGAS–STING pathway to trigger type I interferon responses and antitumour immunity.

Cancer cells operate within a precarious metastable state, which is maintained by the dynamic interplay among RS, CCD and CIN [7] (Figure 5). This RS–CCD–CIN axis enables rapid adaptation while continuously skirting the edge of genomic collapse. At the heart of this system lies a fragile balance between replication fidelity, checkpoint integrity and mitotic accuracy – one that constantly negotiates between survival and entropy.

Persistent RS undermines fork stability and fidelity, which is exacerbated when CCD disrupts checkpoint surveillance. Loss of *TP53* disables the G1/S checkpoint, allowing damaged or underreplicated DNA to progress to mitosis. During this stage, the spindle mediates fragmentation of unresolved replication forks, generating ecDNA and structural rearrangements [101]. Importantly, these ecDNAs can act not only as a consequence of replication and chromosomal stress but also as an active driver within the RS–CCD–CIN network, amplifying RS, titrating replication factors and promoting asymmetric segregation to propagate genomic instability [16,102]. Moreover, CIN-related structures such as micronuclei – resulting from segregation errors – frequently undergo nuclear envelope rupture, promoting DNA DSBs and fork collapse. These events further amplify RS through RAD51-dependent fork reversal and chromothripsis.

Rather than serving as protective measures, stress response pathways are often subverted in this environment. ATR signalling, which is typically activated to stabilize replication forks, becomes dysfunctional under aberrant CDK2 activity in the CCD, leading to premature mitotic entry with unreplicated DNA [103]. Misregulation of Aurora B kinase further stabilizes kinetochore attachment errors, propagating CIN [104]. Moreover, transient relief from RS – such as through RRM2 upregulation in trisomy 8 – disrupts dNTP homeostasis, creating an illusion of genomic stability while masking underlying fragility.

This metastable state is maintained by adaptive stress responses. For example, CIN triggers BUB1–NF-κB signalling to support cell survival, whereas ALKBH5-mediated m^6^A demethylation of BRCA1 transcripts modulates homologous recombination efficiency [105]. TP53 deficiency allows telomeric R-loops to persist into mitosis, where gains in chromosome 3q (e.g. SOX2, PIK3CA) enhance NHEJ, converting RS into heritable structural variants [106,107].

Therapeutic intervention must account for this interconnected axis. Combining ATR inhibitors with MCL1 antagonists can induce ecDNA fragmentation during mitosis, activating the cGAS–STING pathway and triggering antitumour immunity. Rather than targeting individual pathways, therapeutically perturbing the metastable RS–CCD–CIN network to its tipping point may reveal latent vulnerabilities, with ecDNA dynamics acting as both mechanistic readouts and active mediators of genomic destabilization [16,102]. Such interventions must take into account that alternative amplification and structural variation pathways can similarly influence oncogene dosage and stress-buffering capacity, potentially complicating the interpretation of ecDNA-specific effects.

The RS–CCD–CIN axis thus represents a double-edged sword: it enables rapid tumour evolution but depends on tightly regulated instability. Strategically tipping this balance may transform a cancer’s adaptability into its Achilles’ heel.

## Summary and future outlook

The survival strategy of cancer cells relies on their ability to manage and exploit genomic instability. Rather than succumbing to DNA damage, cancer cells exploit RS, CCD and CIN to fuel adaptation and evolution [6]. These mechanisms interact dynamically in a conditionally coupled manner to maintain a metastable state, with the precise balance between entropy and survival emerging from context-dependent feedbacks rather than deterministic causality.

For example, CIN-induced chromosomal breaks can initiate BIR at oncogene loci such as PIK3CA, whereas APOBEC3B activity at ssDNA regions introduces targeted mutations such as TP53 R248W, promoting clonal selection under stress [101,108]. Moreover, ATR signalling – normally protective under RS – is compromised by aberrant CDK2 activity in the CCD, forcing unresolved replication forks into mitosis [109,110]. Spindle tension then fragment these forks into extrachromosomal DNA (ecDNA) amplicons, such as EGFRvIII, enabling escape from targeted therapy [111]. The phenomenon described here as ‘entropy flow redirection’ serves as a descriptive heuristic to summarize observed patterns of instability redistribution, rather than functioning as a defined molecular mechanism with established empirical boundaries.

A key insight into this model lies in revealing the dual-edged nature of oncogene addiction. In ovarian cancer, CCNE1 amplification promotes proliferation but also interferes with CMG helicase activity, aggravating RS [112,113]. Although these tumours are vulnerable to ATR inhibitors, resistant subclones may emerge via ecDNA amplification of genes such as MYC. Resistance is also evident through the rewiring of the DDR network. Notably, the loss of 53BP1 can restore homologous recombination proficiency in BRCA-deficient cells, allowing these cells to bypass reliance on POLθ-mediated repair and prevent metastable collapse [114,115]. Similarly, KRAS-mutant lung cancers exhibit increased origin firing, replication fork collisions and topological stress, creating a synthetic dependence on WEE1 inhibition [116,117]. These contradictions reveal that the very alterations driving tumour growth can also become therapeutic liabilities when their metastable balance is pushed past a threshold.

Future strategies must focus on spatiotemporal characterization and precision disruption of metastable states. Single-cell multiomics (e.g. scRNA-seq + scATAC-seq) has identified distinct subclones – such as CIN-high/RS-low, RS-high/CIN-low and CCD-dominant populations – within the same tumour [118]. Their dynamic interplay contributes to treatment resistance and differential therapeutic responses. In pancreatic cancer, CCD-dominant polyploid cells evade chemotherapy via Hippo–YAP signalling but show metabolic dependence on oxidative phosphorylation, suggesting vulnerability to AMPK inhibitors [119]. Spatial transcriptomics further reveals regional bias: RS markers (e.g. p-RPA32) are enriched at invasive fronts, whereas CIN drivers (e.g. AURKA) are concentrated in tumour cores – patterns influenced by microenvironmental stressors such as hypoxia and ROS [120].

To translate this conceptual framework into precision oncology, we propose the implementation of a ‘dynamic entropy map’ – a stratification algorithm that integrates CCNE1 amplification, which drives basal replicative friction, and Schlafen 11 (SLFN11) expression, an essential effector for drug-induced cytotoxicity. Crucially, to effectively overcome and prevent resistance, therapeutic protocols must evolve from static maintenance to evolutionary steering. We advocate for longitudinal ctDNA surveillance to detect specific ‘entropy flow redirection’ events, such as the restoration of homologous recombination through 53BP1 loss, which typically signals resistance to ATR/PARP inhibitors [121,122]. The detection of such rewiring should trigger a pre-emptive adaptive switch to exploit the resultant collateral sensitivities; for instance, by targeting the newly exposed dependency on POLθ-mediated end-joining [123–125]. This strategy enables clinicians to intercept clonal evolution precisely when the tumour is metabolically and structurally most vulnerable, thereby preventing the establishment of refractory disease.

Clinically, this framework indicates a transition from empirically selected drug combinations to mechanism-informed perturbations. However, the evidence remains preliminary, and the application of metastable collapse strategies is limited by narrow therapeutic indices, patient heterogeneity and temporal constraints on RS. In the NCT05594239 trial, dual inhibition of ATR (berzosertib) and CDK2 (PF-07104091) resulted in a 58% objective response rate in BRCA wild-type breast cancer. Nonetheless, these findings are preliminary and depend on cohort size, trial phase and biomarker selection. Mechanistically, ATR inhibition increases the likelihood of replication fork collapse, while CDK2 inhibition modulates G1/S progression, creating a context-dependent window where mitotic entry may coincide with unrepaired lesions, potentially facilitating ecDNA amplification [38,126]. Conversely, chronic or sustained activation of cGAS–STING signalling can paradoxically support tumour survival and inflammation-associated progression [90]. Persistent signalling may drive NF-κB-mediated transcriptional programs, promoting cytokine-mediated immune evasion, stromal remodelling and compensatory survival pathways [127]. These context-dependent effects underscore the importance of precisely timing therapeutic interventions to exploit acute immunogenic windows while avoiding chronic pro-tumourigenic signalling. In CIN-high tumours, where ongoing CIN continuously generates cytosolic DNA, the balance between acute immunogenic and chronic pro-survival responses is particularly sensitive to dosing schedules, checkpoint modulation and RS management [78,128]. Accordingly, therapeutic strategies that aim to leverage cGAS–STING-mediated vulnerability must integrate the dynamics of DNA fragment accumulation, pathway activation kinetics and cellular context to maximize antitumour efficacy while minimizing inadvertent tumour-promoting effects. Similarly, epigenetic targeting of ecDNA superenhancers, such as through the dCas9–SunTag system recruiting DNMT3A, shows potential to modulate oncogene expression and enhance immune recognition [129], however, these strategies remain largely experimental and require rigorous validation.

Ultimately, the RS–CCD–CIN axis offers a comprehensive framework for understanding cancer plasticity, resistance and treatment failure. These findings highlight that resistance is driven not only by mutations in individual genes but also by a systemic network of entropy regulation. By targeting this metastable architecture, future therapies may progress from static to towards dynamic, entropy-based intervention models. This approach enable facilitate earlier detection of instability, more precise timing of treatment, and improved prediction of patient outcomes. Accordingly, the RS–CCD–CIN axis is not designed to reclassify tumour types or simply rearticulate known instability phenotypes. Instead, it aims to establish a dynamical systems framework for comprehending how cancer cells transition into, sustain and exit metastable evolutionary states.

## Data Availability

Data sharing is not applicable to this article as no data were created or analysed in this study.
